# Supplementary material for: Antibacterial consumption before, during, and after the COVID-19 pandemic in a tertiary care pediatric hospital in Mexico
Source: PLoS One. 2025 Sep 18;20(9):e0329220. doi: 10.1371/journal.pone.0329220 (PMC12445463; doi:10.1371/journal.pone.0329220)
Supplement: S1 Table — † selected model for the prognosis *p < 0.05; ** p < 0.01; *** p < 0.001. (DOCX) [file pone.0329220.s001.docx]

|  | | | | | |
| --- | --- | --- | --- | --- | --- |
| **Parameters** | **Model_arma1** | **Model_arma2** | **Model_arma3^†^** | **Model_arma4** | **Model_arma5** |
| *Constant* | 797.73*** | 798.01*** | 793.68*** | 791.91*** | 790.11*** |
| *Autoregressive* | | | | | |
| *Lag 1* | 0.80*** | 0.91*** | 1.85*** | 0.46*** |  |
| *Lag 2* | 0.09 |  | -0.88*** |  |  |
| *Moving average* | | | | | |
| *Lag 1* | -0.58* | -0.65*** | -1.67*** | 0.29*** |  |
| *Lag 2* |  |  | 0.79*** |  |  |
| *Lag 3* |  |  | -0.18 |  |  |
| *Lag 4* |  |  | 0.15 |  |  |
| *Sigma* | 140.42*** | 140.62*** | 137.60*** | 150.29*** | 157.63*** |
| *Akaike information criteria* | 1308.71 | 1306.98 | 1311.16 | 1318.26 | 1327.82 |
| *Bayesian information criteria* | 1321.83 | 1317.48 | 1332.16 | 1326.14 | 1335.7 |

**S1 Table. Results of the Box-Jenkins second stage comparing parameter estimations for several ARMA models of data from whole hospital**

**^†^** selected model for the prognosis *p<0.05; ** p<0.01; *** p<0.001
