# Supplementary material for: Antibacterial consumption before, during, and after the COVID-19 pandemic in a tertiary care pediatric hospital in Mexico
Source: PLoS One. 2025 Sep 18;20(9):e0329220. doi: 10.1371/journal.pone.0329220 (PMC12445463; doi:10.1371/journal.pone.0329220)
Supplement: Attachment — Submitted filename: Response to reviewers.docx [file pone.0329220.s002.docx]

Dear Editor and Reviewers,

We thank you for the thorough review of our manuscript and for the insightful comments that have helped us improve the quality and clarity of our work. Below, we provide detailed responses to each comment. All language edits are highlighted in yellow, and specific revisions made in response to reviewer comments are highlighted in red in the “Revised Manuscript with Track Changes” document.

**Reviewer #1**

The study reports the evaluation of antibiotic consumption in a pediatric hospital, before, during, and after the greatest activity of the COVID pandemic.
Information is important and data is of interest. However, there are some observations that the authors could address:

1. English needs to be revised, e.g., intensive care unit of neonatology vs neonatal intensive care unit.

We appreciate the reviewer’s observation. We have conducted a comprehensive English language revision throughout the manuscript to improve clarity and consistency. For instance, “intensive care unit of neonatology” was corrected to “neonatal intensive care unit,” and other similar phrases were edited accordingly. A native English speaker from a language editing service reviewed the final version. All changes are highlighted in yellow.

2. There are paragraphs that are not fully understood. E.g., "However, owing to the variability in body weight, the DDD cannot be used directly in the 106 pediatric population as DOT".
Line 78 check PD vs pd.

Thank you for pointing this out. We have revised the unclear sentence to improve comprehension. The sentence now reads: “However, owing to the variability in body weight, the DDD is not a suitable metric for pediatric populations; instead, DOT is recommended.” (Page 6, lines 110-112)

Additionally, we have corrected typographical inconsistencies, including the formatting of "pd" (patient days), which now appears consistently throughout the text.

3. It is not clear what they mean by the study being a secondary analysis. What is the primary analysis?

We thank the reviewer for this important observation. We clarified in the Methods section that this study represents a secondary analysis of hospital administrative data collected for internal monitoring purposes (Pharmacy and Biostatistics Department databases). There was no previous publication or predefined primary study. The original data were collected for internal purposes, and this secondary analysis was specifically designed to retrospectively evaluate trends in antibacterial consumption. (Page 8, lines 163-169).

4. Although the methodology is widely described (perhaps it would even be advisable to evaluate the possibility of synthesizing some sections), the use of the Box-Jenkins model, whose main objective is to forecast, rather than the comparison between past data series, is not clear. This may be important, given that at least visually and in total numbers, it would seem that antibiotic consumption decreased during the pandemic period (especially between the end of 2021 and the beginning of 2023; in this regard, it should also be considered that the greatest impact on the pediatric population was not at the beginning of the pandemic (2020), but a little later).

We appreciate this insightful comment. We clarified in the Methods sections that the Box–Jenkins methodology (ARMA models) was used with the specific aim of forecasting future antibacterial consumption trends. (Page 11, line 226)

We confirm that a time series analysis was performed to identify underlying patterns and to evaluate whether external events, such as the COVID-19 pandemic, had a statistically significant impact on the data series. We applied the Box & Jenkins methodology to model the series, using statistical tests to determine whether the behavior was driven by an underlying pattern or was stochastic, characterized by random and time-independent fluctuations. This analysis also enabled the generation of a forecast for antibiotic consumption based on the identified data structure. While the primary goal of the study was to describe and compare antibacterial consumption across pandemic phases, the forecasting analysis was included as an additional exploration to estimate future consumption based on historical patterns.

4.1 It is possible that there is a higher consumption in this period in the critical units, but a formal statistical comparison does not seem to have been made in this regard, perhaps it should be considered.

Thank you for the observation. In response, we have now included statistical comparisons between the periods (pre-pandemic, pandemic, and post-pandemic) using one-way ANOVA for the hospital-wide data and for the PICUs. These results have been added to the Results section (Pages 11,12, lines 251-253 and Table 1), and are briefly discussed in the Discussion section. (Pages 16-17, lines 329-340)

5. It would be worth commenting on whether carbapenems are not used in the hospital in particular, since there is no mention of this group of antibiotics.

We apologize for this oversight. We have updated the “Background and rationale” and “Methods” sections to explicitly mention that carbapenems (meropenem) were included in the analysis. These antibacterials are subject to prior authorization and are actively monitored by the Infectious Diseases Department. (Page 9, line 185)

6. The discussion seems to have a better order. First, we talk about consumption before and during covid, then we talk about outpatient care in some reports, then we return to covid care in the hospital. Perhaps also consider synthesizing it and focusing it only on the objectives of the study, which is the reporting of antibiotic consumption, and not the discussion of the potential impact of different

stewardship strategies. E.g., more or less from line 350 to 380, the discussion does not seem to be related to the study

We thank the reviewer for the suggestion. The Discussion section has been revised and reorganized to focus more directly on the study objectives: the description and analysis of antibacterial consumption trends. Content unrelated to the main outcomes, particularly regarding stewardship strategies not evaluated in this study (lines 350–380), has been reduced.

**Reviewer #2**

Dear authors. I received the article entitled: Antibacterial consumption before, during, and after the COVID-19 pandemic in a tertiary care pediatric hospital in Mexico for review. Despite the relevance of the manuscript, some points should be clarified as described below:
1. Abstract- Please inform a legend for HIMFG

We have added a legend at the first mention of “HIMFG” in the abstract: “Hospital Infantil de México Federico Gómez (HIMFG)”. (Page 3, line 54)

2. Study design- Please describe exclusion criteria.

We clarified in the Methods section that exclusion criteria included incomplete records (e.g., missing dose, frequency, or administration date) and inconsistent entries (e.g., duplicate records, errors in administration timing). (Page 10, lines 206-211)

3. Please inform if new antibiotics were incorporated during the study period (for example, probably ceftazidime/avibactam was not available in 2016).

We added a statement in the Methods section that none antibacterials were incorporated during the study period. (Page 9, line 187)

4. Please inform if the Pharmacy system was the same during the whole period of if there was some change.

We have clarified that the pharmacy system used to collect antibacterial consumption data remained unchanged throughout the study period, ensuring data consistency. (Page 9, lines 179-180)

5. There's no mention about ethical aspects

We apologize for the omission. We added an Ethical Considerations section stating that the study protocol was reviewed and approved by the institutional Research and Ethics Committee. No patient identifiers were used, and data were analyzed in aggregate. (Page 11, lines 237-242)

6. Discussion. Please explain the reasons for decreasing antibiotic consumption in PICU and Surgical pediatric intensive care unit after the pandemic compared with the previous periods

Thank you for this suggestion. We added a paragraph discussing the likely reasons for decreased antibacterial use in the PICUs after the pandemic, such as the resumption of elective surgeries, fewer COVID-19 admissions, and increased detection of viral infections, leading to more targeted antimicrobial use. (Page 20, lines 409-413)
