# Supplementary material for: Antibacterial consumption before, during, and after the COVID-19 pandemic in a tertiary care pediatric hospital in Mexico
Source: PLoS One. 2025 Sep 18;20(9):e0329220. doi: 10.1371/journal.pone.0329220 (PMC12445463; doi:10.1371/journal.pone.0329220)
Supplement: Attachment — Submitted filename: Response_to_reviewers_auresp_2.docx [file pone.0329220.s003.docx]

Dear Editor and Reviewers,

We sincerely thank the reviewers and the editorial team for their thoughtful and constructive feedback, which has helped us further strengthen our manuscript. Below we provide detailed responses to the remaining comments raised.

We would also like to note that all modifications made in response to reviewer comments are clearly highlighted in blue in the Revised Manuscript with Track Changes.

**Reviewer #1**

**Comment 1:**

“It seems that the authors suggest that the Box & Jenkins model would be a substitute or complement to the analysis of variance. However, the objectives of the two are very different, as are the results: the former focuses on the differences between groups, and the latter on the development of a prediction model. It is worth clarifying this idea further in the discussion.”

**Response:**

We agree with the reviewer’s observation and appreciate the suggestion. We have now clarified in the Methods (**page 10, line 222**) and Discussion section (**page 17, line 343**) that the use of ANOVA and Box-Jenkins responds to different analytical objectives. Specifically, we have added the following sentence:

“It is important to clarify that the ANOVA initially conducted should not be understood as a preliminary step of the Box-Jenkins methodology that we applied later, as both techniques serve very different purposes and were useful for exploring different aspects of our data. While ANOVA provided evidence of differences between means, the Box-Jenkins method allowed us to develop forecasting models.”

This addition reinforces the conceptual distinction and prevents any misinterpretation regarding the relationship between the two methods.

**Comment 2:**

“There is a typo in that section line 338 (comprenhensive vs comprehensive).”

Response:

Thank you for pointing this out. We have corrected the typo from “comprenhensive” to “comprehensive” in **page 17, line 342.**

**Reviewer #2**

**Comment:**
“I’m not still convinced that CAZ-AVI was available in Mexico since 2016. Could you review this information?”

**Response:**
Thank you for this important observation. We have verified the regulatory timeline and clarified the details in the revised manuscript. Ceftazidime-avibactam was approved for use in the general population in Mexico starting in 2015 and subsequently approved for pediatric use in 2019. However, in our specific hospital setting, this antibiotic was only used once, in April 2024, during the study period.

To ensure clarity, we have explicitly stated this in the Methods **(page 9, line 187)** section of the manuscript, as follows:

“Although ceftazidime-avibactam was authorized in Mexico for general use in 2015 and for pediatric use in 2019, in our hospital it was only prescribed in April 2024, and therefore its consumption was minimal.”

We hope this clarification resolves the concern regarding the availability and usage timeline of CAZ-AVI in our study.

**Reference List Review**

In accordance with the journal’s requirements, we reviewed the full reference list. No retracted articles were identified, and no changes to the reference list were necessary.

We hope that these clarifications and revisions satisfactorily address the remaining concerns. We thank the reviewers once again for their constructive feedback and are pleased that the manuscript is now considered technically sound and suitable for publication.
